# Predictors of severity and onset timing of immune-related adverse events in cancer patients receiving immune checkpoint inhibitors: a retrospective analysis

**DOI:** 10.3389/fimmu.2025.1508512

**Published:** 2025-02-18

**Authors:** Qimei Fang, Yan Qian, Zhaolu Xie, Hongqiong Zhao, Yang Zheng, Di Li

**Affiliations:** ^1^ Department of Pharmacy, Second Affiliated Hospital, Chongqing Medical University, Chongqing, China; ^2^ College of Pharmacy, Chongqing Medical University, Chongqing, China

**Keywords:** immune checkpoint inhibitors, immune-related adverse events, predictor, severity, onset time

## Abstract

**Objective:**

To identify predictors of all-grade, grade ≥ 3, and onset time of immune-related adverse events (irAEs) in cancer patients undergoing immune checkpoint inhibitors (ICIs) therapy.

**Methods:**

This retrospective analysis included cancer patients treated with ICIs at Chongqing Medical University Second Affiliated Hospital from 2018 to 2024. Logistic regression and Cox regression analyses were used to identify predictors of all-grade and grade ≥ 3 irAEs and the time of irAE onset.

**Results:**

Among the 3,795 patients analyzed, 1,101 (29.0%) developed all-grade irAEs, and 175 (4.6%) experienced grade ≥ 3 irAEs. Multivariate logistic regression revealed that female (OR = 1.37, p < 0.001), combination therapy (OR = 1.87, p < 0.001), pre-existing autoimmune diseases (AIDs) (OR = 5.15, p < 0.001), pre-existing cirrhosis (OR = 1.34, p = 0.001), antibiotic use during ICIs treatment (OR = 1.51, p < 0.001), and a higher baseline prognostic nutritional index (PNI) (OR = 1.23, p = 0.01) were significant predictors for the development of all-grade irAEs. The predictors for grade ≥ 3 irAEs included age ≥ 60 (OR = 1.49, p = 0.023) and pre-existing AIDs (OR = 2.09, p = 0.005), For the onset time, predictors included female (HR = 1.26, p = 0.001), combination therapy (HR = 1.80, p < 0.001), pre-existing AIDs (HR = 2.25, p < 0.001), and pre-existing infection (HR = 1.20, p = 0.008).

**Conclusions:**

Females, combination therapy, pre-existing AIDs and cirrhosis, antibiotics, and a higher baseline PNI are associated with a higher risk of developing all-grade irAEs. Those aged ≥ 60 and with pre-existing AIDs face a higher risk of severe irAEs. Females, undergoing combination therapy, with pre-existing AIDs and infection generally experience a shorter time to irAEs onset. Multicentric prospective studies are warranted to validate these findings.

## 1 Introduction

Immune checkpoint inhibitors (ICIs) represent a significant advance in cancer treatment, targeting molecules such as programmed cell death 1 (PD-1), programmed cell death ligand-1 (PD-L1), and cytotoxic T-lymphocyte-associated antigen 4 (CTLA-4). By enhancing immune cell activity, ICIs facilitate the destruction of tumor cells and control tumor growth, demonstrating remarkable anti-tumor effects (1). Clinical studies confirm that ICIs, alone or in combination with other therapies, markedly improve cancer treatment outcomes.

However, increasing immune function can sometimes harm normal cells, leading to complex autoimmune and autoinflammatory reactions termed immune-related adverse events (irAEs) (2). As ICIs become increasingly prevalent in clinical use, reports of irAEs have increased. A multicenter retrospective study found that 24% of patients experienced any grade of irAE, while 5.6% experienced grade 3-4 irAEs (3). IrAEs can manifest in any organ, most commonly affecting the skin, endocrine glands, the gastrointestinal tract, the lungs, and the musculoskeletal system (1, 4–6). Although many irAEs can be managed with systemic corticosteroids, severe cases require prompt intervention to prevent potential fatalities (1).

The pathophysiological mechanisms behind irAEs remain largely elusive (1). Current research is often limited to specific diseases (e.g., melanoma (7, 8), non-small cell lung cancer (9, 10), irAEs (e.g., colitis (11), endocrine toxicities (12, 13), or ICIs (e.g., nivolumab (12, 14), pembrolizumab (15). Studies reported that baseline high serum levels of Interleukin-1β (IL-1β), Interleukin-2 (IL-2), and Granulocyte-Macrophage Colony-Stimulating Factor (GM-CSF) are associated with the occurrence of thyroid irAEs (16). Patients with the Human Leukocyte Antigen-death receptor 4 (HLA-DR4) gene are more likely to develop ICIs induced insulin-dependent diabetes (17). Anti-nuclear antibodies (ANA) or rheumatoid factors (RF) may assist in screening for various types of irAEs (18). However, despite the potential value of these biomarkers in predicting irAEs, they are not routinely tested prior to the initiation of ICI therapy. Ideal biomarkers should be suitable for frequent testing, provide rapid results, and be cost-effective. Furthermore, incorporating easily accessible clinical and demographic characteristics enhances prediction accuracy and aids in identifying high-risk patients.

This retrospective cohort study aims to analyze the characteristics of irAEs among patients treated with ICIs and identify predictors of all-grade and severe irAEs and their onset times. This research is intended to serve as a foundation for future large-scale, multi-center studies and to provide robust data to refine clinical treatment strategies and improve the early prevention and identification of irAEs.

## 2 Material and methods

### 2.1 Study population

We retrospectively analyzed cancer patients treated with ICIs at the Second Affiliated Hospital of Chongqing Medical University from January 2018 to January 2024. Eligible participants included patients with clinically and pathologically confirmed malignancies who received ICI therapy, either as monotherapy or in combination with other treatments such as surgery, radiotherapy, or pharmacotherapy. The study excluded patients with more than 20% missing baseline variables, those participating in clinical trials, individuals who received the first dose of ICIs or underwent long-term treatment at other medical institutions, and individuals under 18 years of age (**Supplementary Table 1**).

### 2.2 Data collection

Data were extracted from the Hospital Information System and Laboratory Information Management Systems, including baseline demographics (sex, age, height, and weight), clinical characteristics, and laboratory and imaging data. Comorbidities were defined as diseases diagnosed at baseline, and concomitant medications referred to as those used during ICI therapy. Baseline blood laboratory results, obtained within one week before starting ICI therapy, were used to calculate the platelet-lymphocyte ratio (PLR) and prognostic nutritional index (PNI), the latter calculated as serum albumin plus five times the lymphocyte count. The activities of daily living (ADL) score, numerical rating scale (NRS) score for pain assessment, and nutritional risk screening 2002 (NRS-2002) score were assessed by nurses at the time of patient admission and recorded in the nursing records.

### 2.3 Study assessments

The causal relationship between ICIs and irAEs was categorized using the World Health Organization causality assessment into the following classifications: ‘certain,’ ‘probable,’ ‘possible,’ ‘unlikely,’ ‘unclassified,’ or ‘unclassifiable’ (19). The identification of suspected irAEs was based on the clinical judgment of the attending physician, consultations with specialists, imaging findings, laboratory results, and pathological diagnoses. We assessed the association of the suspected irAEs and categorized them as ‘certain,’ ‘probable,’ or ‘possible’ for inclusion in the study.

The severity of irAEs was assessed using the Common Terminology Criteria for Adverse Events (CTCAE) version 5.0 (20). The onset of irAEs was defined as the interval between initiating ICI therapy and observing abnormal clinical, imaging, or laboratory results indicative of irAEs. Multiple immune-related adverse events (mirAEs) were characterized as the occurrence of two or more irAEs in the same patient, irrespective of their simultaneity. This study received approval from the Ethics Review Committee of the Second Affiliated Hospital of Chongqing Medical University (approval number: (2/2023)-1, approval date: 7, February, 2024).

### 2.4 Statistical analysis

Continuous variables are reported as the median and interquartile range (M[P25-P75]), while categorical variables are expressed as frequency (percentage) N (%). Receiver operating characteristic (ROC) analysis was utilized to derive areas under the curve and optimal cutoff values based on the Youden index. A logistic regression model was applied to identify predictors associated with all grades and grade ≥ 3 irAEs. The timing of irAE onset was analyzed using a Cox proportional hazard model. When a covariate failed to meet the proportional hazards (PH) assumption, a Cox model with time-dependent covariate was implemented. The model assessment involved the Hosmer-Lemeshow goodness-of-fit test to evaluate completeness and predictive accuracy. To maximize statistical power and minimize bias associated with excluding missing data from analyses, multiple imputation was employed to address variables with fewer than 5% missing data, generating five datasets. For missing continuous variables (BMI and laboratory results), imputation was performed using the mean across the datasets. All analyses were repeated with the complete data cohort for comparison. All statistical analyses were performed with IBM SPSS Statistics (version 26.0), and data were visualized with GraphPad Prism 9.0 and Origin2024. Statistical significance was set at p < 0.05.

## 3 Results

### 3.1 Clinical characteristics of patients

In this retrospective study, 4,010 cancer patients received ICIs treatment. After applying exclusion criteria, 215 patients (5.4%) were excluded due to missing more than 20% of baseline data (99 patients), participation in clinical trials (5 patients), received the first dose of ICIs or underwent long-term treatment at other medical institutions (108 patients), and age under 18 years (3 patients), resulting in a final sample of 3,795 patients. **Table 1** details the characteristics of the study population. The median follow-up time after ICI initiation was 22 weeks (IQR: 7 to 52), with males comprising 75% (n = 2,847) of the cohort. The median age was 61 (IQR: 54 to 70), and the median BMI was 22.31 kg/m² (IQR: 20.20 to 24.46). The educational background of the majority was at the secondary level and above (66.4%, n = 2, 520); 32.1% (n = 1,220) were current smokers, and 35.3% (n = 1339) were alcohol consumers. The most common cancers were liver (35.4%, n = 1,342) and lung (29.3%, n = 1,112). At ICI initiation, 74.4% (n = 2,824) of patients had stage III-IV disease, 62.4% (n = 2,368) were partially independent of their daily living activities (ADL = 40-99) and 81.9% (n = 3,108) reported no pain (NRS = 0). Most (40.2%, n = 1,525) had a history of tumor resection surgery, while 10.9% (n = 415) had food, medications, or contrast agents allergies. A total of 76.7% of the patients (n = 2910) had no nutritional risk (NRS 2002 = 0-2).

**Table 1:** General characteristics of patients.

Characteristics		n=3795	Percentage (%)
Demographics, n (%)			
Duration of follow-up from first infusion (weeks)		22 (7-52)	
Sex	Male	2847	75.0
	Female	948	25.0
Age (year), median (IQR)	61 (54-70)		
Age (year)	<60	1609	42.4
	≥ 60	2186	57.6
BMI (kg/m^2^), median (IQR) (Missing =179)		22.31(20.20-24.46)	
BMI (kg/m^2^)	18.5-24.9	2493	65.7
	<18.5	392	10.3
	≥ 25	731	19.3
	Unknown	179	4.7
Educational level	Primary education and below	1275	33.6
	secondary education and above	2520	66.4
Smoker	Never smoked	1956	51.5
	Current smoker	1220	32.1
	Former smoker	619	16.3
Drinker	Never drank	2456	64.7
	Drinker	1339	35.3
Clinical characteristic, n (%)			
Tumor types	Liver	1342	35.4
	Lung	1112	29.3
	Esophagus	213	5.6
	Stomach	146	3.8
	Pancreas	139	3.7
	Others	843	22.2
Disease stage (Missing = 503)	I	203	5.3
	II	265	7.0
	III	992	26.1
	IV	1832	48.3
	Unknown	503	13.3
ADL	100	1364	35.9
	40-99	2368	62.4
	<40	63	1.7
NRS	0	3108	81.9
	1-3	634	16.7
	4-10	53	1.4
NRS 2002 (Missing = 568)	0-2	2910	76.7
	≥ 3	317	8.4
	Unknown	568	15.0
Surgical history	No	2270	59.8
	Yes	1525	40.2
Allergy history	No	3380	89.1
	Yes	415	10.9
aCCI, median (IQR)		7 (5-8)	
PD-1	Sintilimab	1510	39.8
	Camrelizumab	1034	27.2
	Tislelizumab	634	16.7
	Toripalimab	258	6.8
	Pembrolizumab	88	2.3
	Serplulimab	32	0.8
	Zimberelimab	21	0.6
	Nivolumab	19	0.5
	Penpulimab	9	0.2
	Pucotenlimab	7	0.2
PD-L1	Atezolizumab	102	2.7
	Durvalumab	28	0.7
	Adebrelimab	21	0.6
	Sugemalimab	16	0.4
	Envafolimab	4	0.1
PD-1/CTLA-4	Cadonilimab	12	0.3
Treatment program	ICIs	1356	35.7
	ICIs+Targeted therapy	911	24.0
	ICIs+Chemotherapy	1356	35.7
	ICIs+Chemotherapy+Targeted therapy	172	4.5
Comorbidities, n (%)			
Hypertension	Yes	1012	26.7
Diabetes	Yes	626	16.5
AIDs	Yes	127	3.3
Infection	Yes	1131	29.8
Cirrhosis	Yes	970	25.6
HIV	Yes	20	0.5
Concomitant medication, n (%)			
Systemic corticosteroids	Yes	1591	41.9
Immunosuppressant	Yes	34	0.9
Antibacterial	Yes	2651	69.9
Laboratory results, median (IQR)			
ABC (10^9/L)	Missing = 25	0.02 (0.01-0.04)	
AEC (10^9/L)	Missing = 25	0.09(0.04-0.18)	
ALC (10^9/L)	Missing = 27	1.01(0.69-1.36)	
AMC (10^9/L)	Missing = 26	0.43(0.31-0.59)	
ANC (10^9/L)	Missing = 713	3.85(2.69-5.4)	
PLT (10^9/L)	Missing = 28	183(126-252)	
RBC (10^12/L)	Missing = 25	4.05(3.57-4.48)	
WBC (10^9/L)	Missing = 25	5.62(4.18-7.42)	
ALB (g/L)	Missing = 67	39.8(36-43)	

Data are presented as frequencies and percentages for categorical variables, continuous variables were expressed as median and interquartile range M(P25−P75). IQR, interquartile range; NRS, numerical rating scale; NRS2002, nutrition risk screening; aCCI, age-adjusted Charlson Comorbidity Index; AIDs, autoimmune diseases; ADL, Activity of Daily Living; ABC, absolute basophil count; AEC, absolute eosinophil count; ALC, absolute lymphocyte count; AMC, absolute monocyte count; ANC, absolute neutrophil count; PLT, platelet count; RBC, red blood cell count; WBC, white blood cell count; ALB, Albumin; Never smokers, never tried smoking; Current smokers, smoked in the 30 days prior to the survey; Former smokers, currently stopped smoking.

PD-1 inhibitors were administered as follows: Sintilimab was used by 39.8% of the patients (n = 1,510), Camrelizumab by 27.2% (n = 1,034), Tislelizumab by 16.7% (n = 634), Toripalimab by 6.8% (n = 258), Pembrolizumab by 2.3% (n = 88), Serplulimab by 0.8% (n = 32), Zimberelimab by 0.6% (n = 21), Nivolumab by 0.5% (n = 19), Penpulimab by 0.2% (n = 9), and Pucotenlimab also by 0.2% (n = 7). PD-L1 inhibitors included Atezolizumab, used by 2.7% of the patients (n = 102), Durvalumab by 0.7% (n = 28), Adebrelimab by 0.6% (n = 21), Sugemalimab by 0.4% (n = 16), and Envafolimab by 0.1% (n = 4). The PD-1/CTLA-4 inhibitor Cadonilimab was also administered to 0.3% of the cohort (n = 12). The primary treatment modalities were ICIs monotherapy and ICIs combined with chemotherapy, each accounting for 35.7% of the treatments (n = 1,356). Comorbid conditions included hypertension (26.7%, n = 1,012) and diabetes (16.5%, n = 626). During treatment, 69.9% (n = 2,651) of the patients used antibiotics. The median absolute basophil count (ABC) was 0.02 × 10^9^/L (IQR: 0.01 to 0.04), and the median absolute eosinophil count (AEC) was 0.09 × 10^9^/L (IQR: 0.04 to 0.18).

### 3.2 Characteristics of irAEs

In this study, 29.0% of the patients (1,101/3,795) experienced irAEs, and 4.6% (175/3,795) developed severe irAEs of grade ≥ 3. During follow-up, 141 patients experienced mirAEs (range 2-7), resulting in 1,265 documented irAEs. The most common irAEs involved the skin (35.8%, n = 453), followed by endocrine (32.2%, n = 407), liver (7.0%, n = 89), and lung problems (6.4%, n = 81), with the rarest being edema (0.2%, n = 3) and oral complications (0.2%, n = 3). Most irAEs were mild to moderate (grades 1-2, 84.1%, n = 1,064), while 15.9% (n = 201) were classified as severe. Four deaths (0.1%) were attributed to severe interstitial pneumonia (n = 2) or myocarditis (n = 2) (**Table 2**). Specific types of irAEs are shown in **Supplementary Table 2**.

**Table 2:** Characteristics of irAEs.

Types of irAEs	n (%)	Grade					Pharmacotherapy	Steroid therapy	ICIs discontinuation	ICIs rechallenge	IrAEs reactivation
		1	2	3	4	5					
Cutaneous	453 (35.8)	74 (16.3)	301 (66.4)	74 (16.3)	4 (0.9)	0	366 (80.8)	235 (51.9)	70 (15.5)	13 (18.6)	8 (61.5)
Endocrine	407 (32.2)	229 (56.3)	158 (38.8)	19 (4.7)	1 (0.2)	0	250 (61.4)	17 (4.2)	39 (9.6)	14 (35.9)	12 (85.7)
Hepatic	89 (7)	69 (77.5)	11 (12.4)	7 (7.9)	2 (2.2)	0	72 (80.9)	6 (6.7)	10 (11.2)	3 (30)	2 (66.7)
Pulmonary	81 (6.4)	9 (11.1)	48 (59.3)	17 (21)	5 (6.2)	2 (2.5)	64 (79)	55 (67.9)	54 (66.7)	7 (13)	6 (85.7)
Cardiovascular	67 (5.3)	0	45 (67.2)	19 (28.4)	1 (1.5)	2 (3)	60 (89.6)	29 (43.3)	29 (43.3)	2 (6.9)	1 (50)
Gastrointestinal	37 (2.9)	11 (29.7)	18 (48.6)	8 (21.6)	0	0	33 (89.2)	5 (13.5)	9 (24.3)	2 (22.2)	1 (50)
Hematological	30 (2.4)	10 (33.3)	11 (36.7)	5 (16.7)	4 (13.3)	0	24 (80)	2 (6.7)	8 (26.7)	1 (12.5)	1 (100)
Musculoskeletal	29 (2.3)	2 (6.9)	17 (58.6)	7 (24.1)	3 (10.3)	0	25 (86.2)	16 (55.2)	11 (37.9)	0	0
Renal	23 (1.8)	11 (47.8)	10 (43.5)	2 (8.7)	0	0	16 (69.6)	3 (13)	5 (21.7)	0	0
Infusion reaction	18 (1.4)	4 (22.2)	8 (44.4)	4 (22.2)	2 (11.1)	0	17 (94.4)	9 (50)	6 (33.3)	2 (33.3)	2 (100)
Neurologic	15 (1.2)	0	5 (33.3)	8 (53.3)	2 (13.3)	0	15 (100)	11 (73.3)	9 (60)	1 (11.1)	1 (100)
Fatigue	6 (0.5)	3 (50)	3 (50)	0	0	0	2 (33.3)	0	5 (83.3)	1 (20)	0
Pancreatic	4 (0.3)	0	3 (75)	1 (25)	0	0	2 (50)	1 (25)	1 (25)	1 (100)	0
Oral	3 (0.2)	0	3 (100)	0	0	0	3 (100)	3 (100)	1 (33.3)	0	0
Edema	3 (0.2)	0	1 (33.3)	2 (66.7)	0	0	3 (100)	2 (66.7)	2 (66.7)	0	0
Overall	1265 (100)	422 (33.4)	642 (50.8)	173 (13.7)	24 (1.9)	4 (0.3)	952 (75.3)	394 (31.1)	259 (20.5)	47 (18.1)	34 (72.3)

Following the onset of irAEs, most patients (75.3%, 952/1,265) underwent medical treatment (**Supplementary Table 3**), with 31.1% (394/1,265) receiving steroid therapy. For those developing endocrine irAEs, such as hypothyroidism and diabetes, treatments included glucose-lowering medications and hormone replacement therapy. Due to intolerable irAEs, 20.5% of patients (259/1,265) discontinued ICI therapy, mainly due to fatigue (83.3%, 5/6), pneumonitis (66.7%, 54/81), edema (66.7%, 2/3), and neurological issues (60%, 9/15). Of those who discontinued, a small proportion (18.1%, 47/259) later resumed ICI therapy; however, a high recurrence rate of irAEs was observed among them (72.3%, 34/47). Further details are shown in **Table 2**.

**Figure 1:**
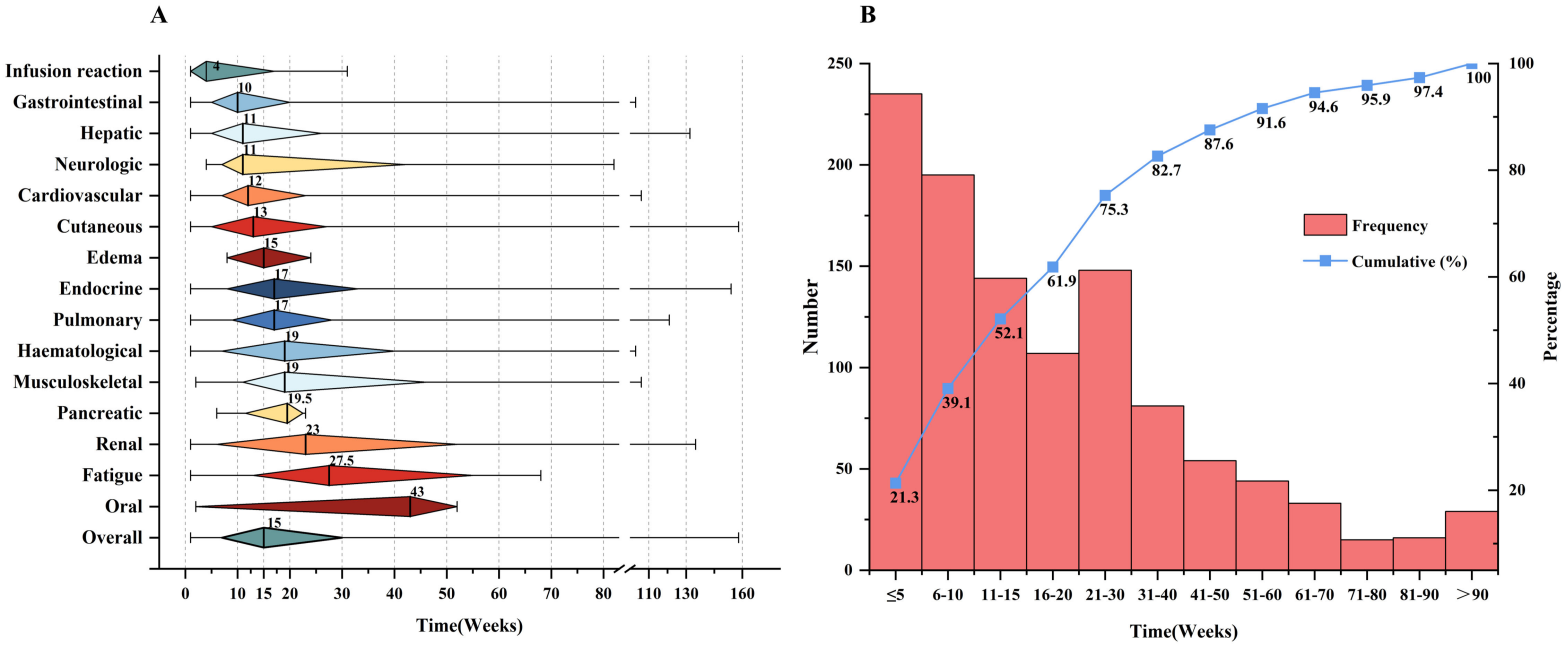
Clinical features of irAEs. **(A)** Time to onset of irAE s since ICIs initiation. The rhombus center lines, rhombus bounds and whiskers denote the medians, first and third quartiles and minimum and maximum values, respectively. **(B)** The number of weeks between ICIs initiation and irAEs diagnosis.

The median time to onset of irAEs was 15 weeks (IQR: 7-30). Infusion reactions, the earliest irAEs, occurred at a median of 4 weeks (IQR: 1-17). This was followed by gastrointestinal irAEs, which appeared at a median of 10 weeks (IQR: 5-20). Oral irAEs were at a median of 43 weeks (IQR: 22.5-47.5), as shown in **Figure 1A**. More than half (52.1%) of patients who experienced irAEs developed their first irAE within the first 15 weeks of starting ICI treatment, with only a minority of cases emerging after 90 weeks. **Figure 1B** shows the number of weeks between ICIs initiation and irAEs diagnosis.

### 3.3 Frequency of irAEs for different ICIs

**Figure 2:**
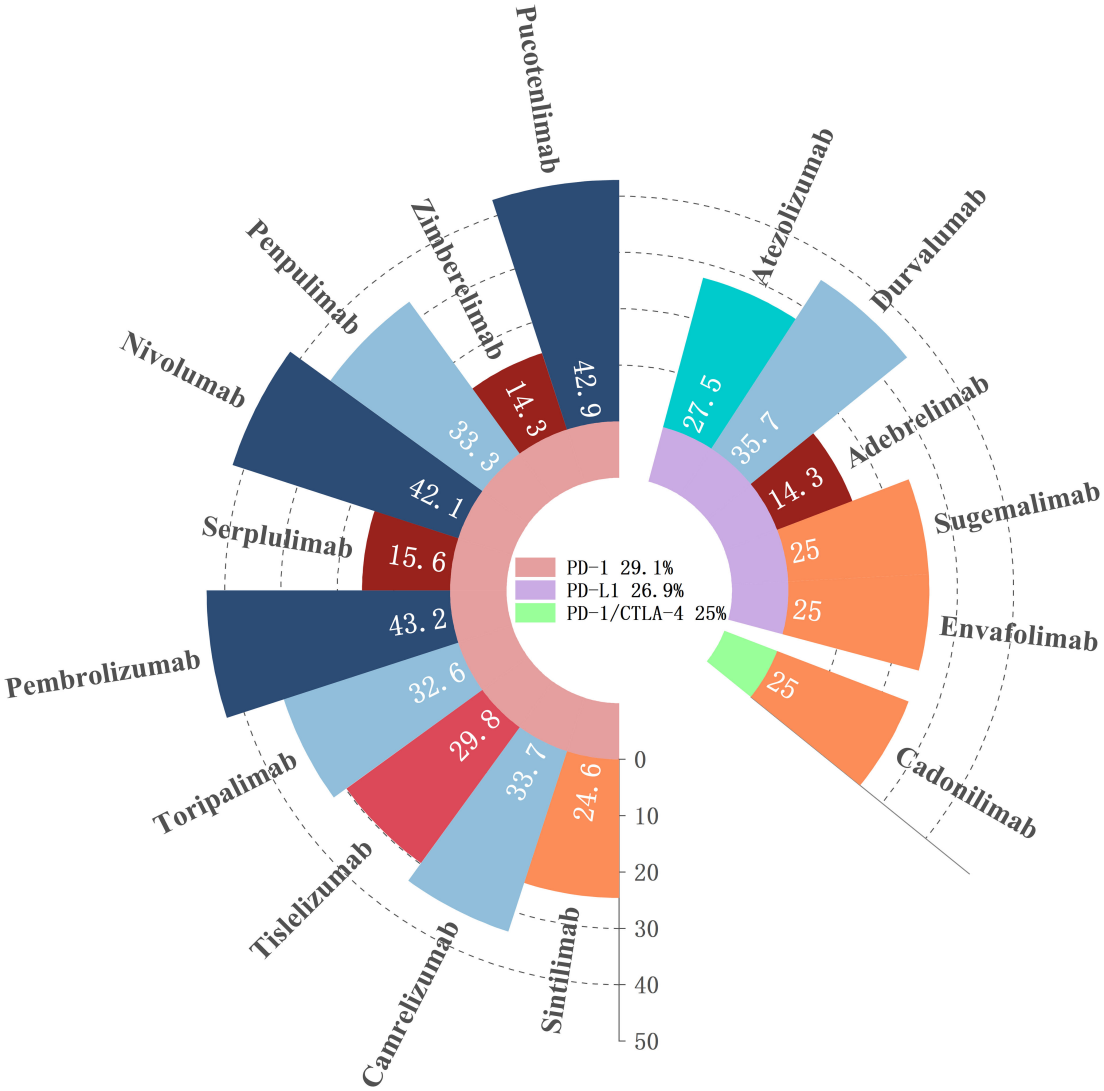
Frequency of irAEs for different ICIs.

The patients were treated with three different types of ICIs, but the incidence of irAEs did not show significant variation between the types: PD-1 vs. PD-L1 (p = 0.531), PD-1 vs. PD-1/CTLA-4 (p = 1.0), and PD-L1 vs. PD-1/CTLA-4 (p = 0.886). The incidence of irAEs associated with PD-1 inhibitors was 29.1%, with specific rates for each drug as follows: Sintilimab at 24.6% (371/1,510), Camrelizumab at 33.7% (348/1,034), Tislelizumab at 29.8% (189/634), Toripalimab at 32.6% (84/258), Pembrolizumab at 43.2% (38/88), Serplulimab at 15.6% (5/32), Nivolumab at 42.1% (8/19), Penpulimab at 33.3% (3/9), and Pucotenlimab at 42.9% (3/7), Zimberelimab at 14.3% (3/21). For PD-L1 inhibitors, the overall irAE incidence was 26.9%, with Atezolizumab at 27.5% (28/102), Durvalumab at 35.7% (10/28), Adebrelimab at 14.3% (3/21), Sugemalimab at 25% (4/16), and Envafolimab at 25% (1/4). Cadonilimab, the PD-1/CTLA-4 inhibitor, had an irAE incidence of 25% (3/12), as detailed in **Figure 2**.

### 3.4 Analysis of predictors of all-grade and grade ≥ 3 irAEs

We categorized participants into two groups: those who experienced immunotherapy-related adverse events (irAE group, n = 1,101, 29.0%) and those who did not (non-irAE group, n = 2,694, 71.0%). The susceptibility factors for irAEs were analyzed using both univariate and multivariate analyses. Univariate analysis identified several significant risk factors for the development of irAEs, including being female (OR = 1.27, p = 0.003), having a BMI ≥ 25 kg/m² (OR = 1.19, p = 0.049), ADL scores (OR = 0.85, p = 0.029), NRS scores (OR = 0.81, p = 0.031), combination therapy (OR = 1.67, p < 0.001), pre-existing autoimmune diseases (AIDs) (OR = 5.09, p < 0.001), pre-existing cirrhosis (OR = 1.21, p = 0.019), antibiotic use during ICI therapy (OR = 1.47, p < 0.001), a higher baseline PNI (optimal cut-off ≥ 43.8) (OR = 1.24, p = 0.004), and a lower baseline PLR (optimal cut-off < 171.6) (OR = 0.80, p = 0.002), detailed in **Table 3**.

**Table 3:** Univariate analysis to determine predictors for incidence and severity of irAEs.

Variables	Category	Univariate analysis				Univariate analysis			
		Non-irAEs (n=2694)	irAEs (n=1101)	OR (95% CI)	*P* value	1-2 Grades (n=926)	3-5Grades (n=175)	OR (95% CI)	*P* value
Demographics, n (%)									
Sex	Male	2057 (76.4)	790 (71.8)	1.27 (1.09-1.49)	**0.003**	669 (72.2)	121 (69.1)	1.16 (0.82-1.65)	0.403
	Female	637 (23.6)	311 (28.2)			257 (27.8)	54 (30.9)		
Age	<60	1147 (42.6)	462 (42.0)	1.03 (0.89-1.18)	0.728	403 (43.5)	59 (33.7)	1.51 (1.08-2.13)	**0.016**
	≥ 60	1547 (57.4)	639 (58.0)			523 (56.5)	116 (66.3)		
BMI (kg/m^2^)	18.5-24.9	1887 (70.0)	754 (68.5)		0.052	644 (69.5)	110 (62.9)		0.153
	<18.5	299 (11.1)	105 (9.5)	0.88 (0.69-1.11)	0.287	88 (9.5)	17 (9.7)	1.13 (0.65-1.97)	0.665
	≥ 25	508 (18.9)	242 (22.0)	1.19 (1.00-1.42)	**0.049**	194 (21.0)	48 (27.4)	1.45 (1.00-2.11)	0.053
Smoker	Never smoked	1376 (51.1)	580 (52.7)		0.667	486 (52.5)	94 (53.7)		0.878
	Current smoker	875 (32.5)	345 (31.3)	0.94 (0.80-1.10)	0.407	293 (31.6)	52 (29.7)	0.92 (0.63-1.33)	0.647
	Former smoker	443 (16.4)	176 (16.0)	0.94 (0.77-1.15)	0.562	147 (15.9)	29 (16.6)	1.02 (0.65-1.61)	0.932
Drinker	Never drank	1721 (63.9)	735 (66.8)	0.88 (0.76-1.02)	0.093	613 (66.2)	122 (69.7)	0.85 (0.60-1.21)	0.366
	Drinker	973 (36.1)	366 (33.1)			313 (33.8)	53 (30.3)		
Clinical characteristic, n (%)									
ADL	100	939 (34.9)	425 (38.6)		0.092	360 (38.9)	65 (37.1)		0.759
	40-99	1710 (63.5)	658 (59.8)	0.85 (0.74-0.98)	**0.029**	550 (59.4)	108 (61.7)	1.09 (0.78-1.52)	0.624
	<40	45 (1.7)	18 (1.6)	0.88 (0.51-1.55)	0.665	16 (1.7)	2 (1.1)	0.69 (0.16-3.08)	0.629
NRS	0	2186 (81.1)	922 (83.7)		0.071	778 (84.0)	144 (82.3)		0.146
	1-3	473 (17.6)	161 (14.6)	0.81 (0.66-0.98)	**0.031**	136 (14.7)	25 (14.3)	0.99 (0.63-1.58)	0.977
	4-10	35 (1.3)	18 (1.6)	1.22 (0.69-2.16)	0.498	12 (1.3)	6 (3.4)	2.70 (1.00-7.31)	0.051
Treatment program	monotherapy	1051 (39.0)	305 (27.7)	1.67 (1.43-1.95)	**<0.001**	252 (27.2)	53 (30.3)	0.86 (0.60-1.23)	0.405
	Combination therapy	1643 (61.0)	796 (72.3)			674 (72.8)	122 (69.7)		
Surgical history	Yes	1067 (39.6)	458 (41.6)	1.09 (0.94-1.25)	0.256	385 (41.6)	73 (41.7)	1.01 (0.72-1.40)	0.973
Allergy history	Yes	278 (10.3)	137 (12.4)	1.24 (0.99-1.54)	0.057	114 (12.3)	23 (13.1)	1.08 (0.67-1.74)	0.760
aCCI		7 (5-8)	7 (5-8)	1.01 (0.98-1.05)	0.437	7 (5-8)	7 (6-8)	1.02 (0.94-1.10)	0.654
Comorbidities, n (%)									
AIDs	Yes	43 (1.6)	84 (7.6)	5.09 (3.50-7.41)	**<0.001**	61 (6.6)	23 (13.1)	2.15 (1.29-3.57)	**0.003**
Cirrhosis	Yes	660 (24.5)	310 (28.2)	1.21 (1.03-1.41)	**0.019**	266 (28.7)	44 (25.1)	0.83 (0.58-1.21)	0.334
Infection	Yes	787 (29.2)	344 (31.2)	1.10 (0.95-1.28)	0.214	279 (30.1)	65 (37.1)	1.37 (0.98-1.92)	0.067
HIV	Yes	16 (0.6)	4 (0.4)	0.61 (0.20-1.83)	0.378	4 (0.4)	0 (0.0)	0.00 (0.00-)	0.999
Concomitant medication, n (%)									
Antibacterial	Yes	1821 (67.6)	830 (75.4)	1.47 (1.25-1.72)	**<0.001**	693 (74.8)	137 (78.3)	1.21 (0.82-1.79)	0.332
Immunosuppressant	Yes	24 (0.9)	10 (0.9)	1.02 (0.49-2.14)	0.959	8 (0.9)	2 (1.1)	1.33 (0.28-6.30)	0.722
Laboratory results, median (IQR)									
AEC (10^9/L)		0.09 (0.04-0.18)	0.10 (0.05-0.19)	1.02 (0.81-1.30)	0.849	0.10 (0.05-0.18)	0.10 (0.05-0.22)	1.23 (0.61-2.47)	0.564
ABC (10^9/L)		0.02 (0.01-0.04)	0.02 (0.01-0.04)	0.17 (0.01-4.71)	0.298	0.02 (0.01-0.04)	0.02 (0.01-0.04)	0.34 (0.00-1240.05)	0.794
AMC (10^9/L)		0.43 (0.32-0.59)	0.43 (0.30-0.59)	0.86 (0.65-1.14)	0.292	0.43 (0.30-0.58)	0.44 (0.29-0.66)	1.62 (0.87-3.02)	0.131
RBC (10^12/L)		4.04 (3.55-4.48)	4.06 (3.62-4.51)	1.07 (0.97-1.18)	0.186	4.08 (3.65-4.52)	3.95 (3.50-4.49)	0.85 (0.68-1.08)	0.183
WBC (10^9/L)		5.66 (4.22-7.42)	5.49 (4.13-7.40)	0.99 (0.97-1.02)	0.579	5.49 (4.06-7.26)	5.70 (4.41-8.06)	1.05 (1.00-1.10)	**0.029**
PNI	≥ 43.8	1554 (57.7)	691 (62.8)	1.24 (1.07-1.43)	**0.004**	583 (63.0)	108 (61.7)	0.95 (0.68-1.32)	0.755
PLR	≥ 171.6	1449 (53.8)	532 (48.3)	0.80 (0.70-0.92)	**0.002**	443 (47.8)	89 (50.9)	1.13 (0.82-1.56)	0.464

Data are presented as frequencies and percentages for categorical variables, continuous variables were expressed as median and interquartile range M(P25−P75). NRS, numerical rating scale; aCCI, age-adjusted Charlson Comorbidity Index; AIDs, autoimmune diseases; ADL, Activity of Daily Living; AEC, absolute eosinophil count; ABC, absolute basophil count; AMC, absolute monocyte count; RBC, red blood cell count; WBC, white blood cell count; PNI, prognostic nutritional index; PLR, platelet-lymphocyte ratio; Never smokers, never tried smoking; Current smokers, smoked in the 30 days prior to the survey; Former smokers, currently stopped smoking. Logistic regression models were used to analyze predictors of both the incidence and severity of irAEs.

Bold values signify p < 0.05.

**Figure 3:**
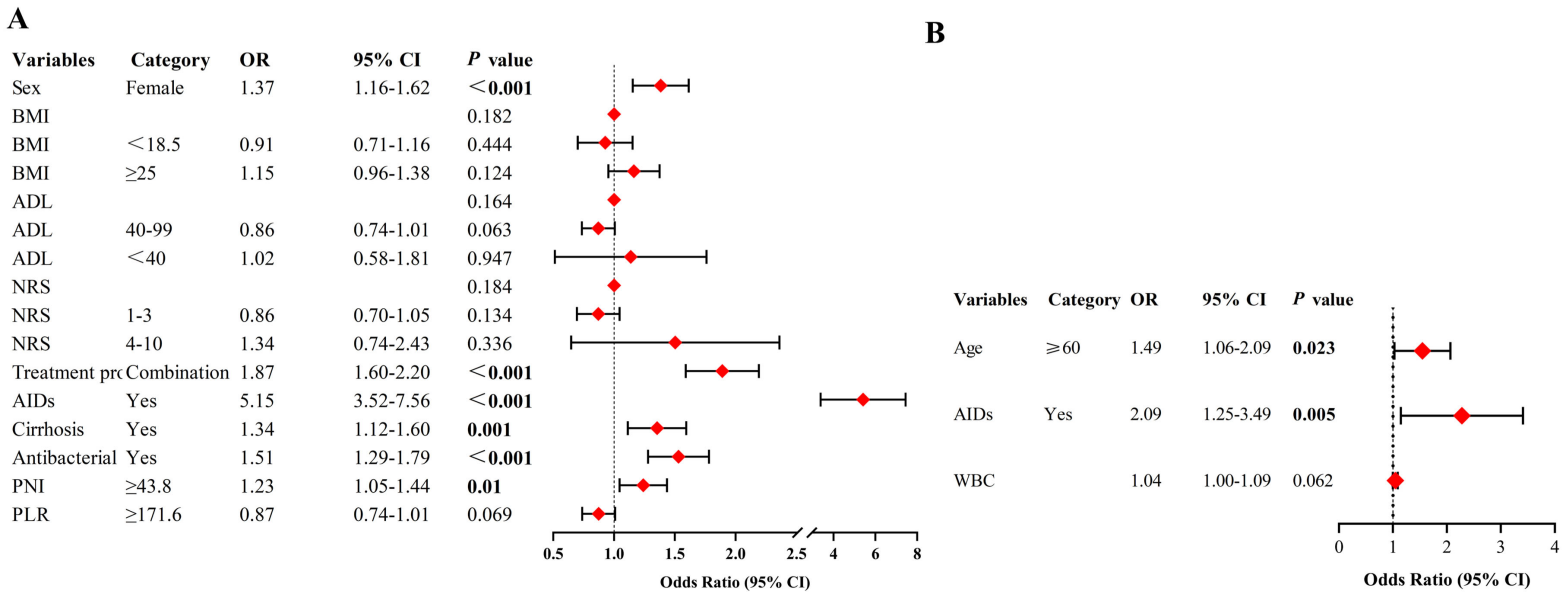
**(A)** Multivariate analysis to determine predictors for incidence of irAEs. **(B)** Multivariate analysis to determine predictors for severity of irAEs. NRS, numerical rating scale; ADL, Activity of Daily Living; AIDs, autoimmune diseases; PNI, prognostic nutritional index; PLR, platelet-lymphocyte ratio; WBC, white blood cell count; OR, odds ratio; CI, confidence interval; Never smokers, never tried smoking; Current smokers, smoked in the 30 days prior to the survey; Former smokers, currently stopped smoking. Logistic regression models were used to analyze predictors of both the incidence and severity of irAEs.

Multivariate analysis demonstrated that being female (OR = 1.37, 95% CI: 1.16-1.62, p < 0.001), undergoing combination therapy (OR = 1.87, 95% CI: 1.60-2.20, p < 0.001), having pre-existing AIDs (OR = 5.15, 95% CI: 3.52-7.56, p < 0.001), pre-existing cirrhosis (OR = 1.34, 95% CI: 1.12-1.60, p = 0.001), and the use of antibiotics during ICI treatment (OR = 1.51, 95% CI: 1.29-1.79, p < 0.001), as well as a higher PNI (optimal cut-off ≥ 43.8) (OR = 1.23, 95% CI: 1.05-1.44, p = 0.01), were independent predictors of irAEs. Factors such as BMI, ADL, NRS, and PLR did not significantly correlate with an increased risk of irAEs (**Figure 3A**).

Patients with irAEs were divided into groups: those with grade 1-2 irAEs (84.1%, n = 926) and those with grade 3-5 irAEs (15.9%, n = 175). A comparative analysis of the variables between these groups was conducted. Univariate analysis identified age ≥ 60 (OR = 1.51, p = 0.016), pre-existing AIDs (OR = 2.15, p = 0.003), and higher baseline white blood cell count (WBC) levels (OR = 1.05, p = 0.029) as significant predictors of irAE severity (**Table 3**). Multivariate analysis further confirmed that age ≥ 60 (OR = 1.49, 95% CI: 1.06-2.09, p = 0.023) and pre-existing AIDs (OR = 2.09, 95% CI: 1.25-3.49, p = 0.005) were independent predictors for higher-grade irAEs (**Figure 3B**).

### 3.5 Analysis of the predictors of the onset time of irAEs

Univariate analysis using the Cox PH model and time-dependent Cox regression model identified several factors as significant predictors for earlier onset of irAEs: being female (HR = 1.25, p = 0.001), ADL scores < 40 (HR = 1.64, p = 0.039), having a NRS score of 4-10 (HR = 1.63, p = 0.04), combination therapy (HR = 1.81, p < 0.001), pre-existing AIDs (HR = 2.34, p < 0.001), pre-existing infection (HR = 1.23, p = 0.002), higher baseline absolute monocyte count (AMC) levels (HR = 1.33, p = 0.013), and higher baseline WBC levels (HR = 1.02, p = 0.029). Subsequent multivariate analysis confirmed that being female (HR = 1.26, 95% CI: 1.10-1.44, p = 0.001), undergoing combination therapy (HR = 1.80, 95% CI: 1.57-2.05, p < 0.001), having pre-existing AIDs (HR = 2.25, 95% CI: 1.80-2.81, p < 0.001), and pre-existing infection (HR = 1.20, 95% CI: 1.05-1.37, p = 0.008) were independent predictors for a faster onset of irAEs. These findings are detailed in **Table 4**.

**Table 4:** Univariate and multivariate analysis to determine predictors of the onset time of irAEs.

Variables	Category	Univariate analysis			Multivariate analysis		
		HR	95% CI	*P* value	HR	95% CI	*P* value
Demographics							
Sex	Female	1.25	1.09-1.42	**0.001**	1.26	1.10-1.44	**0.001**
Age*	≥ 60	1.00	1.00-1.01	0.359			
BMI				0.835			
BMI	<18.5	1.06	0.87-1.31	0.549			
BMI	≥ 25	1.01	0.87-1.17	0.889			
Smoker	Never smoked			0.706			
Smoker	Current smoker	0.95	0.84-1.09	0.491			
Smoker	Former smoker	0.95	0.80-1.12	0.513			
Drinker*	Drinker	1.00	0.99-1.00	0.394			
Clinical characteristic							
ADL	100			0.105			0.184
ADL	40-99	1.05	0.93-1.19	0.417	0.98	0.86-1.11	0.716
ADL	<40	1.64	1.03-2.64	**0.039**	1.52	0.94-2.44	0.086
NRS	0			0.067			0.226
NRS	1-3	1.11	0.94-1.31	0.231	1.06	0.90-1.26	0.489
NRS	4-10	1.63	1.02-2.60	**0.040**	1.48	0.92-2.36	0.105
Treatment program	Combination therapy	1.81	1.59-2.07	**<0.001**	1.80	1.57-2.05	**<0.001**
Surgical history*	Yes	1.00	1.00-1.01	0.145			
Allergy history*	Yes	1.00	1.00-1.01	0.671			
Comorbidities							
AIDs	Yes	2.34	1.87-2.92	**<0.001**	2.25	1.80-2.81	**<0.001**
Cirrhosis*	Yes	1.00	1.00-1.01	0.217			
Infection	Yes	1.23	1.08-1.39	**0.002**	1.20	1.05-1.37	**0.008**
HIV	Yes	0.70	0.26-1.87	0.478			
Concomitant medication							
Antibacterial*	Yes	1.00	1.00-1.01	0.178			
Immunosuppressant*	Yes	1.00	0.98-1.01	0.939			
Laboratory results							
ABC (10^9/L)		0.70	0.26-1.87	0.478			
AEC (10^9/L)		1.02	0.85-1.23	0.794			
AMC (10^9/L)		1.33	1.06-1.67	**0.013**	1.19	0.92-1.55	0.186
RBC (10^12/L)		0.96	0.88-1.04	0.305			
WBC (10^9/L)		1.02	1.00-1.04	**0.029**	1.01	0.99-1.03	0.572
PNI	≥ 43.8	1.01	0.89-1.14	0.873			
PLR	≥ 171.6	1.00	0.88-1.12	0.948			

NRS, numerical rating scale; AIDs, autoimmune diseases; ADL, Activity of Daily Living; ABC, absolute basophil count; AEC, absolute eosinophil count; AMC, absolute monocyte count; RBC, red blood cell count; WBC, white blood cell count; PLR, platelet-lymphocyte ratio; PNI, prognostic nutritional index; HR, hazard ratio; CI, confidence interval. Never smokers, never tried smoking; Current smokers, smoked in the 30 days prior to the survey; Former smokers, currently stopped smoking. Cox regression and Cox proportional hazards model with time-dependent covariates was employed to assess the predictors related to the timing of irAEs onset. *Using time-dependent cox regression model.

Bold values signify p < 0.05.

### 3.6 Sensitivity analyses

The missing data proportion across variables ranged from 0.7% to 4.7%, with 3,556 cases providing complete data for all key variables. IrAEs were observed in 28.6% of patients, including 4.6% who developed severe irAEs (grade ≥ 3). The distribution of variables with missing data was comparable between the imputed dataset and the observed complete case dataset (**Supplementary Table 4**). The results of the multivariable regression analysis using complete case data were generally consistent with those from the imputed dataset (**Supplementary Tables 5**, **6**, **Supplementary Figure 1**). In the complete case dataset, multivariable regression analysis showed that NRS (4–10) was significantly associated with irAE severity (OR=3.26, 95% CI: 1.17–9.11, p=0.024). In contrast, univariable analysis of the imputed dataset revealed a near-significant association (OR=2.70, 95% CI: 1.00–7.31, p=0.051).

## 4 Discussion

### 4.1 Characteristics of irAEs

The incidence of irAEs varies depending on cancer types, the category of ICIs, and patient-related factors. A recent meta-analysis, included clinical trials of ICIs in the treatment of unresectable hepatocellular carcinoma, reported an overall irAEs incidence of 31.1% and 6.6% for grade ≥ 3 irAEs (21). In addition, Shankar B et al. reported that 33.1% of patients with non-small cell lung cancer treated with anti-PD(L) experienced irAEs of all grades (22). In pan-cancer patients, a meta-analysis reported that the incidence of PD-1 inhibitors induced all-grade irAEs was 26.82%, and 6.1% for grade ≥ 3 irAEs (23). Another single-center retrospective study finding that 39.05% of patients experienced irAEs of all grades, and 9.5% for grade ≥ 3 irAEs (24). Another meta-analysis of clinical trials reported that in ICIs monotherapy, the incidence of overall irAEs was higher in CTLA-4 inhibitors (53.8%) compared to PD-L1 (17.1%) or PD-1 inhibitors (26.5%), and CTLA-4 inhibitors were also more likely to cause severe irAEs (25). Moreover, CTLA-4 inhibitors commonly cause colitis, pituitary inflammation, and rashes, whereas PD-(L)1 inhibitors were more likely to casuse pneumonia, hypothyroidism, arthralgia and vitiligo (26). In our study, the all-grade irAE incidence was 29.0% and grade ≥ 3 irAEs was 4.6%, which were similar to or slightly lower than the results reported above. The most common irAEs were related to the skin, endocrine system, liver and pulmonary. As for the reasons, we supposed that some mild (grade 1-2) irAEs might be underestimated or overlooked by the hospital information system; since our median follow-up time was 22 (IQR 7-52) weeks, some potential late-onset irAEs might not be recorded; that nobody was administrated with CTLA-4 inhibitors in our cohort, may lead to the lower incidence of overall, severe irAEs and gastrointestinal events.

It’s worth noting that 4.6% patients experienced grade ≥ 3 grade irAEs, indicating the relatively safe of ICIs. And most toxic effects were reversible and improved after discontinuation of ICI therapy and/or administration of corticosteroids. However, 4 patients died due to pneumonia (n = 2) and myocarditis (n = 2), highlighting the need for enhanced monitoring of patients during treatment, with particular emphasis on the early identification and intervention of life-threatening irAEs. In present study, 12 patients were treated with Cadonilimab, a PD-1/CTLA-4 bispecific antibody, which was demonstrated the lowest incidence of irAEs at 25%. IrAEs caused by ICIs are associated with the recruitment of immune cells bearing Fc receptors. Cadonilimab was designed to remove Fc receptor binding and effector functions, thereby improving its efficacy and safety (27). However, considering the small number of patients treated with the PD-1/CTLA-4 bispecific antibody, a larger sample size may be required to draw statistically significant conclusions.

### 4.2 Predictors of irAEs

Our findings suggest that women have a significantly higher risk of developing irAEs when treated with ICIs. However, the role of sex differences remains debated. A meta-analysis did not find a significant impact of gender sex on irAE occurrence (28), while other studies reported that women are more prone to endocrine irAEs, particularly thyroid dysfunction, and men to hypophysitis (29). This suggests that sex may influence the type of irAE. Additional research has proposed that female is a potential predictor for irAEs (30). Hormonal differences, particularly high estrogen levels, may enhance immune responses by increasing proinflammatory cytokines and amplifying T-helper cell responses, potentially explaining the increased risk of irAEs in women (31, 32). Similar to large randomized controlled trials of immunotherapy, there was a relatively small number of female participants in present study. This might introduce bias into the results. Thus further research is needed to clarify the relationship between sex hormones and irAEs.

While the increasing use of combination strategies might improve the efficacy of cancer immunotherapy but could also amplify irAEs (10, 33, 34). In our study, the incidence of irAEs increased significantly when ICIs were combined with chemotherapy or targeted therapies. Therefore, exploring combination therapies to maximize benefits while minimizing irAEs is crucial. Hyperthermia was found to enhance ICI efficacy and reduced irAEs, likely by improving ICI and immune cell aggregation and tumor chemotaxis (35, 36). Nanoparticle-based thermotherapy enhances treatment targeting by creating a more favorable environment for immunotherapy (37). Two-dimensional nanomaterials further improve photo-thermal therapy, drug delivery, and reduce toxicity, offering new support for tumor immunotherapy (38).

Patients with pre-existing AIDs face an elevated risk of irAEs. Although ICIs show promise in these populations, they are typically excluded from clinical trials. However, evidence is accumulating regarding the use of ICIs in this ‘at-risk’ population (8, 10, 39, 40). Therefore, patients with pre-existing AIDs should be closely monitored to mitigate the risk of irAEs during ICI therapy. Pre-existing cirrhosis was another significant risk factor for irAEs. Cirrhosis-related immune dysfunction leads to systemic immunodeficiency and inflammation. The immunodeficiency results from the disruption of local immune function in the liver and systemic immune cell dysfunction. While inflammation is reflected by an increased production of pro-inflammatory cytokines (41). The systemic symptoms of cirrhosis may affect the response to ICI treatment, but the underlying mechanisms have not been reported. Additionally, many symptoms caused by extrahepatic diseases associated with cirrhosis may be difficult to distinguish from irAEs but can synergistically worsen organ function, potentially leading to an overdiagnosis of irAEs (42). Our study preliminarily uncovered the relationship between cirrhosis and irAEs, but further validation is needed.

A significant association was found between antibiotic exposure, both after the initiation of ICIs and before the onset of irAEs, and the occurrence of irAEs. This association was also observed with antibiotic exposure prior to ICI treatment (43). It was found that patients who received antibiotics after initiating ICI therapy and before the onset of immune-mediated diarrhea or colitis (IMDC) had a significantly higher incidence of IMDC compared to those who were exposed to antibiotics either before or both before and after ICI treatment (P < 0.001) (44). Disruptions in microbial diversity, particularly affecting neutrophil and T-cell activation pathways, are linked to higher irAE incidence (43). On the other hand, emerging research suggests that the gut microbiome may help predict irAEs. A random forest classifier with 14 microbial features demonstrated strong discriminatory power between non-irAE and irAE patients (AUC = 0.88) (45). Another single-center prospective study revealed significant differences in the gut microbiome composition between patients with non-irAE and those with mild or severe irAE (46). Bifidobacterium longum and Lactobacillus sp. were found to enhance ICI efficacy and mitigate toxic reactions, while Lachnospiraceae spp. and Streptococcus spp. were linked to the development of irAEs (47). These studies indicate that the reduction in microbial diversity caused by antibiotic use may increase the risk of irAEs. Therefore, antibiotic use in ICI-treated cancer patients should be carefully evaluated to mitigate the potential risks.

Routine peripheral blood markers such as PLR and PNI have been associated with irAEs. Unlike classical inflammatory mediators such as IL-6, IL-8, and IL-17 (48), these markers are easily accessible, cost-effective, and stable in clinical settings. PNI, which combines albumin levels and lymphocyte counts, reflects a patient’s inflammatory and immune status (49). In advanced cancer, increased inflammation leads to increased neutrophils, reduced lymphocytes, and lower albumin levels, which can suppress cancer immunity and diminish ICI efficacy, possibly explaining the lower irAE incidence in patients with reduced PNI (50). Our study found that a PNI ≥ 43.8 was associated with a higher irAE risk, supported by other studies identifying high PNI as a predictor of irAE development (50, 51). Baseline PNI could be a useful tool for early irAE identification, potentially reducing hospitalization and treatment costs.

Univariate analysis also identified a BMI ≥ 25 as a risk factor for irAEs. Studies have shown that overweight and obese individuals (BMI ≥ 30) are more likely to develop irAEs compared to those with normal BMI (52, 53). Although lower PLR levels were associated with irAEs in the literature (48), these were not independent risk factors in our study. More research is needed to validate these findings, as confounders may have influenced the results.

### 4.3 Severe irAEs

Although numerous studies have found a positive correlation between irAEs and treatment efficacy (54), severe irAEs can be life-threatening if not properly managed. Current guidelines recommend discontinuing treatment, either temporarily or permanently, when Grade 3 or 4 irAEs occur. Our study found that age ≥ 60 years and pre-existing AIDs were associated with severe irAEs. Retrospective analyses show that older patients are more likely to experience fatal irAEs, with a significant age difference between those with severe outcomes (median age 70 vs. 62 years; p = 0.009) (55). Patients aged ≥ 70 also have higher rates of Grade 3-4 toxicities (56). Analysis of 17,006 lung cancer patients from the FDA’s FAERS database revealed older patients not only experience more irAEs but also face higher toxicity levels (57), likely due to reduced CD8+ T cell counts associated with immune senescence (57, 58).

Pre-existing AIDs significantly increase both the risk and severity of irAEs. A prospective study found that patients with AIDs had higher odds ratios to develop any grade of irAEs (OR = 1.91) and severe irAEs (OR = 1.44) (8). These patients are also more likely to discontinue anti-PD-1 therapy due to toxicity (59). Therefore, close monitoring of elderly patients, those with AIDs is crucial to effectively managing severe irAEs.

NRS was demonstrated a significant stratification effect (NRS 4-10) in the complete case dataset (p = 0.024), although there is currently limited direct evidence supporting this association. We hypothesize that the continued use of NSAIDs or opioids for pain management may mask early symptoms of certain irAEs, such as mild arthritis, myalgia, or abdominal pain, potentially delaying diagnosis and increasing the risk of more severe irAEs. However, no significant association was observed in the imputed dataset. This discrepancy may be attributed to changes in the sample distribution within the imputed dataset, particularly the higher proportion of the low-risk group (NRS 0-3), which likely diluted the effect of NRS in the univariate analysis (p = 0.051). This suggests that the impact of NRS may vary across different datasets, potentially reflecting a weaker effect or one influenced by sample characteristics. Further research is needed to confirm this hypothesis.

### 4.4 Timing of irAEs

IrAEs can affect almost any organ system and may occur at any point during treatment, even months after the last ICI administration (60). Due to this unpredictability, our study aimed to identify predictors of irAE occurrence. The results indicate that females not only have a higher risk of developing irAEs but also tend to develop them earlier than males, which may be attributed to stronger immune responses in females (31).

Combination therapy with ICIs and chemotherapy or targeted therapies was associated with earlier irAE onset. A meta-analysis showed that combination therapy led to a shorter median time to all-grade irAE onset compared to nivolumab monotherapy (6.0 weeks vs. 8.2 weeks, p < 0.001) (61).

Patients with pre-existing AIDs also exhibited earlier irAE onset (8), consistent with our findings. Additionally, patients with pre-existing infections (bacterial, viral, or fungal) experienced a significantly earlier onset of irAEs. This phenomenon may be closely linked to the effects of chronic infections on the immune system. Studies suggest that chronic infections can induce T-cell exhaustion through the expression of immune checkpoints like PD-1 (62). The use of ICI may restore the immune response against pathogens, triggering inflammatory reactions to latent or chronic infections, thereby increasing the risk of irAEs (63), However, current research directly supporting the relationship between infections and the timing of irAEs is limited. Our study is a preliminary exploration, further research is needed to confirm this finding and investigate the underlying biological mechanisms.

## 5 Limitations

This single-center retrospective study has inherent limitations, including potential regional and informational biases. Firstly, Due to the large sample size, some variables were statistically significant but such significance in clinical practice may be limited, such as higher baseline PNI (OR = 1.23) and pre-existing infection (HR = 1.20). Secondly, the data collection based on the hospital information system may lead to underestimate and overlook the minor irAEs, while relatively short follow-up time (median [IQR], 22[7-52] weeks) may incompletely record some potential late-onset irAEs. These inevitably brought the lower incidence of overall, severe irAEs. Thirdly, the association between efficacy and irAEs was not explored due to the patients lost to follow-up or incomplete medical records, resulting in the sample size with appreciable and accurate efficacy was limited to further analysis so far. Finally, while multiple imputations were used to handle missing data, this may still introduce some bias. Therefore, multi-center further retrospective studies and prospective studies are necessary to validate our findings and gain a broader understanding of irAEs in diverse populations.

## 6 Strengths

This study has several strengths. First, it features a large sample size with broad inclusion criteria, reflecting real-world clinical scenarios and improving the generalizability of the findings. Second, it covers a wide range of irAEs, addressing organ-specific and systemic adverse events, providing a comprehensive view of ICI-associated complications. Finally, this is one of the first real-world studies to explore predictors of irAE onset timing, addressing an important gap in the literature and offering valuable information for future research.

## 7 Conclusions

Females, combination therapy, pre-existing AIDs and cirrhosis, antibiotics, and a higher baseline PNI are associated with a higher risk of developing all-grade irAEs. Those aged ≥ 60 and with pre-existing AIDs face a higher risk of severe irAEs. Females, undergoing combination therapy, with pre-existing AIDs and infection generally experience a shorter time to irAEs onset. Multicentric prospective studies are warranted to validate these findings.

## Data Availability

The raw data supporting the conclusions of this article will be made available by the authors, without undue reservation.
