# Supplementary material for: Predictors of severity and onset timing of immune-related adverse events in cancer patients receiving immune checkpoint inhibitors: a retrospective analysis
Source: Front Immunol. 2025 Feb 18;16:1508512. doi: 10.3389/fimmu.2025.1508512 (PMC11876122; doi:10.3389/fimmu.2025.1508512)
Supplement: Supplementary file 1 [file DataSheet1.docx]

**Supplementary Table 1.** Inclusion and exclusion criteria.

| **Inclusion** | **Exclusion** |
| --- | --- |
| 1．Patients with clinically and pathologically confirmed malignancies. | 1．Patients with more than 20% missing baseline variables |
| 2．Received ICI therapy, either as monotherapy or in combination with other treatments (e.g., surgery, radiotherapy, or pharmacotherapy) | 2．Participants in clinical trials |
|  | 3. Received the first dose of ICIs or underwent long-term treatment at other medical institutions |
|  | 4．Under 18 years |

**Supplementary Table 2**. Summary of irAEs

| **Types of irAEs** | **Count** | **Types of irAEs** | **Count** | **Percentage** |
| --- | --- | --- | --- | --- |
| Cutaneous | 453 |  |  |  |
|  |  | Rash | 285 | 22.5 |
|  |  | Pruritus | 82 | 6.5 |
|  |  | Reactive cutaneous capillary endothelial proliferation | 79 | 6.2 |
|  |  | Bullous pemphigoid | 6 | 0.5 |
|  |  | Drug-induced epidermolysis bullosa acquisita | 1 | 0.1 |
| Endocrine | 407 |  |  |  |
|  |  | Thyroid dysfunction | 380 | 30.0 |
|  |  | Adrenal insufficiency | 13 | 1.0 |
|  |  | Hyperglycemia | 10 | 0.8 |
|  |  | Hypophysitis | 2 | 0.2 |
|  |  | Parathyroid dysfunction | 2 | 0.2 |
| Hepatic | 89 |  |  |  |
|  |  | Hepatitis | 89 | 7.0 |
| Pulmonary | 81 |  |  |  |
|  |  | Pneumonia | 81 | 6.4 |
| Cardiovascular | 67 |  |  |  |
|  |  | Myocarditis | 66 | 5.2 |
|  |  | Vasculitis | 1 | 0.1 |
| Gastrointestinal | 37 |  |  |  |
|  |  | Diarrhea | 26 | 2.1 |
|  |  | Colitis | 10 | 0.8 |
|  |  | Proctitis | 1 | 0.1 |
| Haematological | 30 |  |  |  |
|  |  | Thrombocytopenia | 27 | 2.1 |
|  |  | Pancytopenia | 2 | 0.2 |
|  |  | Venous thrombosis | 1 | 0.1 |
| Musculoskeletal | 29 |  |  |  |
|  |  | Arthritis | 13 | 1.0 |
|  |  | Myositis | 16 | 1.3 |
| Renal | 23 |  |  |  |
|  |  | Kidney injury | 23 | 1.8 |
| Infusion reaction | 18 |  |  |  |
|  |  | Infusion reaction | 18 | 1.4 |
| Neurologic | 15 |  |  |  |
|  |  | Peripheral neuropathy | 6 | 0.5 |
|  |  | Encephalitis | 3 | 0.2 |
|  |  | Meningitis | 3 | 0.2 |
|  |  | Myasthenia gravis | 2 | 0.2 |
|  |  | Optic neuritis | 1 | 0.1 |
| Fatigue | 6 |  |  |  |
|  |  | Fatigue | 6 | 0.5 |
| Pancreatic | 4 |  |  |  |
|  |  | Pancreatitis | 3 | 0.2 |
|  |  | Elevated amylase | 1 | 0.1 |
| Oral | 3 |  |  |  |
|  |  | Oral Mucositis | 3 | 0.2 |
| Edema | 3 |  |  |  |
|  |  | Facial and bilateral ankle edema | 1 | 0.1 |
|  |  | Bilateral lower limb edema | 2 | 0.2 |

**Supplementary Table 3.** Medications for the treatment of irAEs.

| **Types of irAEs** | **Pharmacotherapy** |
| --- | --- |
|  |  |
| Cutaneous | Methylprednisolone, Prednisone, Dexamethasone, Mometasone Furoate Cream, Halometasone/Triclosan Cream, Compound Camphor Cream, Compound Flumetasone Ointment, Mupirocin Ointment, Calamine Lotion, Loratadine, Olopatadine Hydrochloride,Cetirizine, Ebastine, Epinastine ,Vitamin C , Fufang Huangbai Ye, Compound Glycyrrhizin |
| Endocrine | Thyroxine, Bisoprolol, Propranolol, Thiamazole, Metoprolol Tartrate, Insulin, Hydrocortisone, Prednisone, Metformin Hydrochlorid, Sitagliptin Phosphate |
| Hepatic | Methylprednisolone, Prednisolone, Glutathione, Ursodeoxycholic Acid, Magnesium Isoglycyrrhizinate, Bicyclol, Compound Glycyrrhizin, Tiopronin, Silibinin |
| Pulmonary | Methylprednisolone, Prednisone, Cyclophosphamide, Dexamethasone |
| Cardiovascular | Methylprednisolone, Prednisone, Creatine Phosphate Sodium, Trimetazidine, Sodium Fructose Diphosphate, Coenzyme Q10 |
| Gastrointestinal | Methylprednisolone, Prednisone, Dexamethasone, Mesalazine, Loperamide, Live Combined Bacillus Subtilis and Enterococcus Faecium Enteric-coated, Omeprazole, Live Combined Bifidobacterium，Lactobacillus and Enterococcus, Montmorillonite powder |
| Haematological | Prednisone, Dexamethasone, Recombinant Human Thrombopoietin, ShengXueXiaoBanJiaoNang, Recombinant Human Erythropoietin, ShengXueNingPian, Recombinant Human Granulocyte Colony-stimulating Factor, Recombinant Human Granulocyte Colony-Stimulating Factor, Avatrombopag Maleate, Rivaroxaban |
| Musculoskeletal | Prednisone, Methylprednisolone, Dexamethasone, Tripterysium Glycosides, Creatine Phosphate Sodium, Flurbiprofen Axetil, Loxoprofen, Celecoxib, Betamethasone |
| Renal | Prednisone, Methylprednisolone, NiaoDuQingKeLi, Piperazine Ferulate, JinShuiBao, Bailing Tablets |
| Infusion reaction | Dexamethasone, Methylprednisolone, Loratadine |
| Neurologic | Prednisone, Dexamethasone, Methylprednisolone, Mecobalamin, Vitamin B1, Citicoline |
| Pancreatic | Methylprednisolone |
| Oral | Methylprednisolone, Recombinant Human Epidermal Growth Factor |
| Edema | Prednisone, Methylprednisolone, Spironolactone |

**Supplementary Table 4.** Distributions of variables in complete case data and datasets after multiple imputation.

| **Characteristics** | **Category** | **Number (%) with missing data** | **Complete case**  **(n=3556)** | **Multiple imputation**  **(N=3795)** |
| --- | --- | --- | --- | --- |
| Demographics, n (%) | | | | |
| Sex | Male | 0 | 2687(75.6) | 2847(75.0) |
|  | Female | 0 | 869(24.4) | 948(25.0) |
| Age (year), median (IQR) |  | 0 | 61(54-70) | 61(54-70) |
| BMI (kg/m^2^) |  | 179(4.7) | 22.31(20.20-24.49) | 22.27(20.20-24.44) |
| Smoker | Never smoked | 0 | 1834(51.6) | 1956(51.5) |
|  | Current smoker | 0 | 1146(32.2) | 1220(32.1) |
|  | Former smoker | 0 | 576(16.2) | 619(16.3) |
| Drinker | Drinker | 0 | 1251(35.2) | 1339(35.3) |
| Clinical characteristic, n (%) | | | | |
| ADL | 100 | 0 | 1280(36.0) | 1364(35.9) |
|  | 40-99 | 0 | 2219(62.4) | 2368(62.4) |
|  | ＜40 | 0 | 57(1.6) | 63(1.7) |
| NRS | 0 | 0 | 2926(82.3) | 3108(81.9) |
|  | 1-3 | 0 | 581(16.3) | 634(16.7) |
|  | 4-10 | 0 | 49(1.4) | 53(1.4) |
| Treatment program | Combination therapy | 0 | 2311(65.0) | 2439(64.3) |
| Surgical history | Yes | 0 | 1434(40.3) | 1525(40.2) |
| Allergy history | Yes | 0 | 397(11.2) | 415(10.9) |
| aCCI | Yes | 0 | 7（5-8） | 7（5-8） |
| Comorbidities, n (%) | | | | |
| AIDs | Yes | 0 | 119(3.3) | 127(3.3) |
| Cirrhosis | Yes | 0 | 919(25.8) | 970(25.6) |
| Infection | Yes | 0 | 1062(29.9) | 1131(29.8) |
| HIV | Yes | 0 | 18(0.5) | 20(0.5) |
| Concomitant medication, n (%) | | | | |
| Antibacterial | Yes | 0 | 2478(69.7) | 2651(69.9) |
| Immunosuppressant | Yes | 0 | 29(0.8) | 34(0.9) |
| Laboratory results, median (IQR) | | | | |
| ABC (10^9/L) |  | 25(0.7) | 0.02(0.01-0.04) | 0.02(0.01-0.04) |
| AEC (10^9/L) |  | 25(0.7) | 0.10(0.05-0.18) | 0.09(0.04-0.18) |
| ALC (10^9/L) |  | 27(0.7) | 1.02(0.69-1.36) | 1.02(0.69-1.36) |
| AMC (10^9/L) |  | 26(0.7) | 0.43(0.31-0.59) | 0.43(0.31-0.59) |
| PLT (10^9/L) |  | 28(0.7) | 184(127-252) | 183(127-252) |
| RBC (10^12/L) |  | 25(0.7) | 4.06(3.59-4.49) | 4.05(3.58-4.48) |
| WBC (10^9/L) |  | 25(0.7) | 5.62(4.19-7.40) | 5.62(4.20-7.41) |
| ALB (g/L) |  | 67(1.8) | 39.9(36.2-43) | 39.8(36-42.9) |

**Supplementary Table 5.** Univariate analysis based on complete case data to determine predictors for incidence and severity of irAEs.

| **Variables** | **Category** | **Univariate analysis** | | | | **Univariate analysis** | | | |
| --- | --- | --- | --- | --- | --- | --- | --- | --- | --- |
|  |  | **Non-irAEs**  **(n=2539)** | **irAEs**  **(n=1017)** | **OR (95% CI)** | ***P* value** | **1-2 Grades**  **(n=854)** | **3-5 Grades**  **(n=163)** | **OR (95% CI)** | ***P* value** |
| Demographics, n (%) | | | | | | | | | |
| Sex | Male | 1951(76.8) | 736(72.4) | 1.27(1.07-1.49) | **0.005** | 620(72.6) | 116(71.2) | 1.07(0.74-1.56) | 0.708 |
|  | Female | 588(23.2) | 281(27.6) | |  | 234(27.4) | 47(28.8) |  |  |
| Age | ＜60 | 1072(42.2) | 437(43.0) | 0.97(0.84-1.12) | 0.683 | 382(44.7) | 55(33.7) | 1.59(1.12-2.26) | **0.010** |
|  | ≥ 60 | 1467(57.8) | 580(57.0) |  |  | 472(55.3) | 108(66.3) |  |  |
| BMI (kg/m2) | 18.5-24.9 | 1761(69.4) | 687(67.6) |  | **0.016** | 587(68.7) | 100(61.3) |  | 0.167 |
|  | ＜18.5 | 291(11.5) | 96(9.4) | 0.85(0.66-1.08) | 0.183 | 79(9.3) | 17(10.4) | 1.26(0.72-2.22) | 0.418 |
|  | ≥ 25 | 487(19.2) | 234(23.0) | 1.23(1.03-1.47) | **0.023** | 188(22.0) | 46(28.2) | 1.44(0.98-2.11) | 0.066 |
| Smoker | Never smoked | 1296(51.0) | 538(52.9) | | 0.603 | 452(52.9) | 86(52.8) |  | 0.973 |
|  | Current smoker | 828(32.6) | 318(31.3) | 0.93(0.79-1.09) | 0.352 | 266(31.1) | 52(31.9) | 1.03(0.71-1.50) | 0.888 |
|  | Former smoker | 415(16.3) | 161(15.8) | 0.93(0.76-1.15) | 0.523 | 136(15.9) | 25(15.3) | 0.97(0.59-1.57) | 0.889 |
| Drinker | Never drank | 1624(64.0) | 681(67.0) | 0.88(0.75-1.02) | 0.091 | 568(66.5) | 113(69.3) | 0.88(0.61-1.26) | 0.484 |
|  | Drinker | 915(36.0) | 336(33.0) |  |  | 286(33.5) | 50(30.7) |  |  |
| Clinical characteristic, n (%) | | | | | | | | | |
| ADL | 100 | 885(34.9) | 395(38.8) |  | 0.081 | 333(39.0) | 62(38.0) |  | 0.925 |
|  | 40-99 | 1612(63.5) | 607(59.7) | 0.84(0.73-0.98) | **0.027** | 506(59.3) | 101(62.0) | 1.07(0.76-1.51) | 0.693 |
|  | ＜40 | 42(1.7) | 15(1.5) | 0.80(0.44-1.46) | 0.468 | 15(1.8) | 0(0.0) | 0.00(0.00-) | 0.998 |
| NRS | 0 | 2067(81.4) | 859(84.5) |  | **0.030** | 723(84.7) | 136(83.4) |  | 0.109 |
|  | 1-3 | 440(17.3) | 141(13.9) | 0.77(0.63-0.95) | **0.013** | 120(14.1) | 21(12.9) | 0.93(0.57-1.53) | 0.776 |
|  | 4-10 | 32(1.3) | 17(1.7) | 1.28(0.71-2.31) | 0.417 | 11(1.3) | 6(3.7) | 2.90(1.05-7.97) | **0.039** |
| Treatment program | monotherapy | 969(38.2) | 276(27.1) | 0.60(0.51-0.71) | **＜0.001** | 229(26.8) | 47(28.8) | 0.90(0.62-1.31) | 0.595 |
|  | Combination therapy | 1570(61.8) | 741(72.9) |  |  | 625(73.2) | 116(71.2) | |  |
| Surgical history | Yes | 1006(39.6) | 428(42.1) | 1.11(0.96-1.28) | 0.176 | 358(41.9) | 70(42.9) | 1.04(0.74-1.46) | 0.808 |
| Allergy history | Yes | 263(10.4) | 134(13.2) | 1.31(1.05-1.64) | **0.016** | 111(13.0) | 23(14.1) | 1.10(0.68-1.78) | 0.700 |
| aCCI |  | 7(5-8) | 7 (5-8) | 1.01(0.97-1.05) | 0.699 | 7(5-8) | 7(5-8) | 1.02(0.94-1.11) | 0.627 |
| Comorbidities, n (%) | | | | | | | | | |
| AIDs | Yes | 40(1.6) | 79(7.8) | 5.26(3.57-7.75) | **＜0.001** | 58(6.8) | 21(12.9) | 2.03(1.19-3.45) | **0.009** |
| Cirrhosis | Yes | 624(24.6) | 295(29.0) | 1.25(1.07-1.48) | **0.006** | 252(29.5) | 43(26.4) | 0.86(0.59-1.25) | 0.420 |
| Infection | Yes | 741(29.2) | 321(31.6) | 1.12(0.96-1.31) | 0.161 | 262(30.7) | 59(36.2) | 1.28(0.90-1.82) | 0.166 |
| HIV | Yes | 15(0.6) | 3(0.3) | 0.50(0.14-1.72) | 0.271 | 3(0.4) | 0(0.0) | 0(0-) | 0.999 |
| Concomitant medication, n (%) | | | | | | | | | |
| Immunosuppressant | Yes | 20(0.8) | 9(0.9) | 1.13(0.51-2.48) | 0.771 | 8(0.9) | 1(0.6) | 0.65(0.81-5.26) | 0.689 |
| Antibacterial | Yes | 1712(67.4) | 766(75.3) | 1.47(1.25-1.74) | **＜0.001** | 640(74.9) | 126(77.3) | 1.14(0.76-1.70) | 0.522 |
| Laboratory results, median (IQR) | | | | | | | | | |
| ABC (10^9/L) | | 0.02(0.01-0.04) | 0.02(0.01-0.04) | 0.22(0.01-6.25) | 0.372 | 0.02(0.01-0.04) | 0.02(0.01-0.04) | 0.37(0.00-1615.95) | 0.818 |
| AEC (10^9/L) | | 0.09(0.04-0.18) | 0.10(0.05-0.20) | 1.06(0.83-1.34) | 0.656 | 0.10(0.05-0.19) | 0.10(0.06-0.22) | 1.32(0.66-2.62) | 0.430 |
| AMC (10^9/L) | | 0.43(0.32-0.59) | 0.43(0.30-0.59) | 0.87(0.66-1.16) | 0.355 | 0.43(0.30-0.58) | 0.44(0.29-0.67) | 1.77(0.92-3.39) | 0.085 |
| RBC (10^12/L) | | 4.05(3.56-4.48) | 4.07(3.65-4.53) | 1.10(0.99-1.22) | 0.073 | 4.09(3.66-4.53) | 4.02(3.53-4.52) | 0.88(0.69-1.12) | 0.300 |
| WBC (10^9/L) | | 5.68(4.22-7.42) | 5.49(4.13-7.36) | 0.99(0.97-1.02) | 0.512 | 5.49(4.09-7.24) | 5.46(4.37-8.06) | 1.04(0.99-1.09) | 0.082 |
| PNI | ≥43.8 | 1470(57.9) | 652(64.1) | 1.30(1.12-1.51) | **0.001** | 550(64.4) | 102(62.6) | 0.92(0.65-1.31) | 0.656 |
| PLR | ≥171.6 | 1365(53.8) | 484(47.6) | 0.78(0.68-0.90) | **0.001** | 402(47.1) | 82(50.3) | 1.14(0.81-1.59) | 0.449 |

Data are presented as frequencies and percentages for categorical variables, continuous variables were expressed as median and interquartile range M(P25−P75). Definition of abbreviations: NRS, numerical rating scale; aCCI, age-adjusted Charlson Comorbidity Index; AIDs, autoimmune diseases; ADL, Activity of Daily Living; AEC, absolute eosinophil count; ABC, absolute basophil count; AMC, absolute monocyte count; RBC, red blood cell count; WBC, white blood cell count; PNI, prognostic nutritional index; PLR, platelet-lymphocyte ratio; Never smokers, never tried smoking; Current smokers, smoked in the 30 days prior to the survey; Former smokers, currently stopped smoking. Logistic regression models were used to analyze predictors of both the incidence and severity of irAEs. Bold values signify p < 0.05.


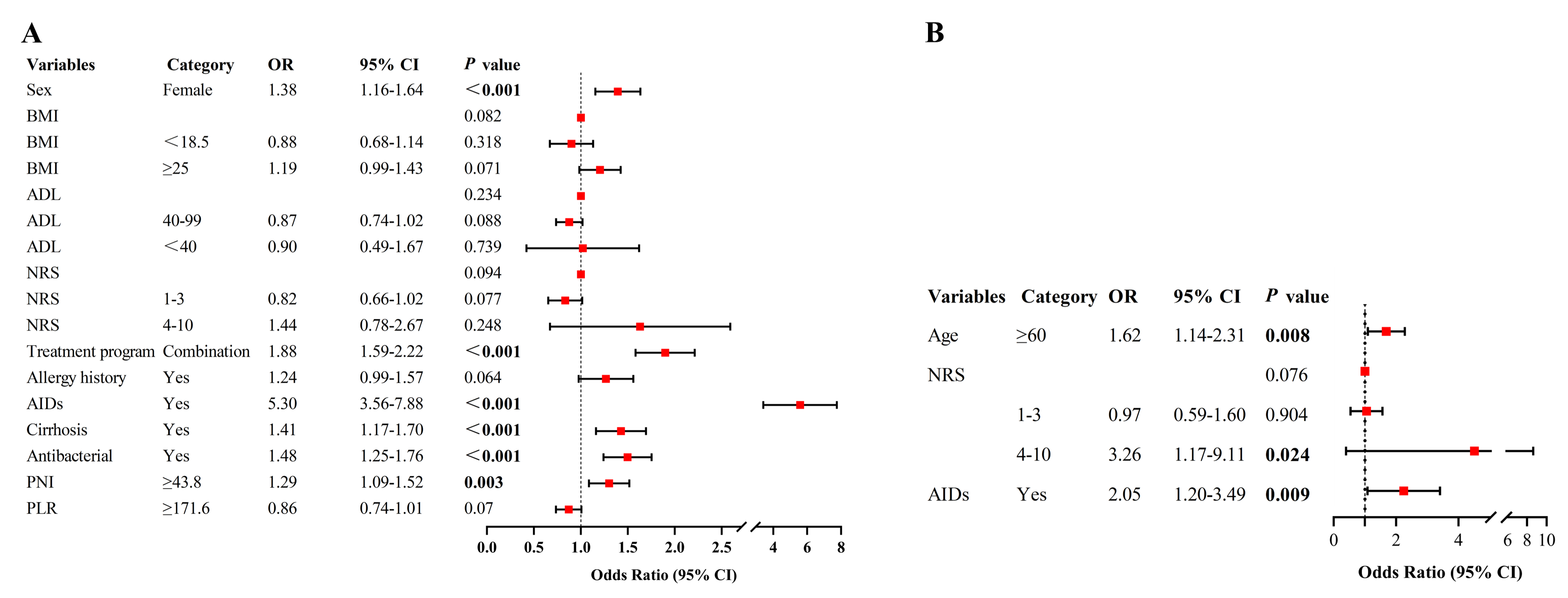


**Supplementary** **Figure 1.** **(A)** Multivariate analysis based on complete case data to determine predictors for incidence of irAEs. **(B)** Multivariate analysis based on complete case data to determine predictors for severity of irAEs.

Definition of abbreviations: NRS, numerical rating scale; ADL, Activity of Daily Living; AIDs, autoimmune diseases; PNI, prognostic nutritional index; PLR, platelet-lymphocyte ratio; WBC, white blood cell count; OR, odds ratio; CI, confidence interval; Never smokers, never tried smoking; Current smokers, smoked in the 30 days prior to the survey; Former smokers, currently stopped smoking. Logistic regression models were used to analyze predictors of both the incidence and severity of irAEs. Bold values signify p < 0.05

**Supplementary Table 6.** Univariate and multivariate analysis based on complete case data to determine predictors of the onset time of irAEs.

| **Variables** | **Category** | **Univariate analysis** | | | **Multivariate analysis** | | |
| --- | --- | --- | --- | --- | --- | --- | --- |
|  |  | **HR** | **95% CI** | ***P* value** | **HR** | **95% CI** | ***P* value** |
| Demographics |  |  |  |  |  |  |  |
| Sex | Female | 1.23 | 1.07-1.41 | **0.003** | 1.25 | 1.09-1.44 | **0.002** |
| Age* | ≥ 60 | 1.00 | 1.00-1.00 | 0.486 |  |  |  |
| BMI |  |  |  | 0.812 |  |  |  |
| BMI | ＜18.5 | 1.04 | 0.84-1.29 | 0.695 |  |  |  |
| BMI | ≥ 25 | 1.04 | 0.90-1.21 | 0.567 |  |  |  |
| Smoker | Never smoked |  |  | 0.626 |  |  |  |
| Smoker | Current smoker | 0.94 | 0.82-1.08 | 0.415 |  |  |  |
| Smoker | Former smoker | 0.94 | 0.78-1.12 | 0.461 |  |  |  |
| Drinker* | Drinker | 1.00 | 0.99-1.00 | 0.350 |  |  |  |
| Clinical characteristic | |  |  |  |  |  |  |
| ADL | 100 |  |  | 0.281 |  |  |  |
| ADL | 40-99 | 1.05 | 0.93-1.19 | 0.445 |  |  |  |
| ADL | ＜40 | 1.48 | 0.89-2.48 | 0.134 |  |  |  |
| NRS | 0 |  |  | 0.086 |  |  | 0.195 |
| NRS | 1-3 | 1.08 | 0.90-1.29 | 0.410 | 1.04 | 0.87-1.24 | 0.707 |
| NRS | 4-10 | 1.67 | 1.03-2.70 | **0.036** | 1.55 | 0.96-2.51 | 0.074 |
| Treatment program | Combination therapy | 1.85 | 1.61-2.13 | **＜0.001** | 1.83 | 1.59-2.11 | **＜0.001** |
| Surgical history* | Yes | 1.00 | 1.00-1.01 | 0.138 |  |  |  |
| Allergy history* | Yes | 1.00 | 1.00-1.01 | 0.572 |  |  |  |
| Comorbidities | | | | | | | |
| AIDs | Yes | 2.34 | 1.86-2.94 | **＜0.001** | 2.24 | 1.78-2.82 | **＜0.001** |
| Cirrhosis* | Yes | 1.00 | 1.00-1.01 | 0.111 |  |  |  |
| Infection | Yes | 1.24 | 1.09-1.42 | **0.001** | 1.22 | 1.07-1.39 | **0.004** |
| HIV | Yes | 0.63 | 0.20-1.96 | 0.427 |  |  |  |
| Concomitant medication | | | | | | | |
| Antibacterial* | Yes | 1.00 | 1.00-1.01 | 0.170 |  |  |  |
| Immunosuppressant* | Yes | 1.00 | 0.98-1.01 | 0.960 |  |  |  |
| Laboratory results |  |  |  |  |  |  |  |
| ABC (10^9/L) |  | 0.76 | 0.05-11.34 | 0.845 |  |  |  |
| AEC (10^9/L) |  | 1.05 | 0.88-1.24 | 0.613 |  |  |  |
| AMC (10^9/L) |  | 1.34 | 1.06-1.69 | **0.016** | 1.23 | 0.97-1.56 | 0.081 |
| RBC (10^12/L) |  | 0.98 | 0.90-1.07 | 0.684 |  |  |  |
| WBC (10^9/L) |  | 1.02 | 1.00-1.04 | 0.050 |  |  |  |
| PNI | ≥ 43.8 | 1.06 | 0.94-1.21 | 0.344 |  |  |  |
| PLR | ≥ 171.6 | 0.97 | 0.86-1.10 | 0.615 |  |  |  |

Definition of abbreviations: NRS, numerical rating scale; AIDs, autoimmune diseases; ADL, Activity of Daily Living; ABC, absolute basophil count; AEC, absolute eosinophil count; AMC, absolute monocyte count; RBC, red blood cell count; WBC, white blood cell count; PLR, platelet-lymphocyte ratio; PNI, prognostic nutritional index; HR, hazard ratio; CI, confidence interval. Never smokers, never tried smoking; Current smokers, smoked in the 30 days prior to the survey; Former smokers, currently stopped smoking. Cox regression and Cox proportional hazards model with time-dependent covariates was employed to assess the predictors related to the timing of irAEs onset. *: Using time-dependent cox regression model. Bold values signify p < 0.05.
